# Prognostic factors for survival with nab-paclitaxel plus gemcitabine in metastatic pancreatic cancer in real-life practice: the ANICE-PaC study

**DOI:** 10.1186/s12885-018-5101-3

**Published:** 2018-11-29

**Authors:** Ana Fernández, Mercedes Salgado, Adelaida García, Elvira Buxò, Ruth Vera, Jorge Adeva, Paula Jiménez-Fonseca, Guillermo Quintero, Cristina Llorca, Mamen Cañabate, Luis Jesús López, Andrés Muñoz, Patricia Ramírez, Paula González, Carlos López, Margarita Reboredo, Elena Gallardo, Manuel Sanchez-Cánovas, Javier Gallego, Carmen Guillén, Nuria Ruiz-Miravet, Víctor Navarro-Pérez, Juan De la Cámara, Inmaculada Alés-Díaz, Roberto Antonio Pazo-Cid, Alberto Carmona-Bayonas

**Affiliations:** 1 Complejo Hospitalario Universitario Ourense, Calle Ramon Puga Noguerol, 54, 32005 Ourense, Spain; 2 Complejo Universitario Ourense, Ourense, Spain; 3 Institut Català d’Oncologia (ICO) Hospital Dr. Trueta, Girona, Spain; 4 Hospital Clínic de Barcelona, Barcelona, Spain; 5 Complejo Hospitalario de Navarra, Navarra, Spain; 6 Hospital 12 de Octubre, Madrid, Spain; 7 Hospital Universitario Central de Asturias, Asturias, Spain; 8 Hospital Lucus Agustí, Lugo, Spain; 9 Hospital de Elda, Alicante, Spain; 10 Hospital Público Lluis Alcanyis de Xátiva, Xátiva, Spain; 11 Hospital Virgen de la Salud, Toledo, Spain; 12 Hospital Gregorio Marañón, Madrid, Spain; 13 Hospital Puerta del Mar, Cadiz, Spain; 14 Hospital Universitario de Vigo, Vigo, Spain; 15 Hospital Marqués de Valdecilla, Santander, Spain; 16 Hospital A Coruña Teresa Herrera, A Coruña, Spain; 17 Hospital de Pontevedra, Pontevedra, Spain; 18 Hospital Morales Meseguer, Murcia, Spain; 19 Hospital de Elche, Elche, Spain; 20 Hospital Ramón y Cajal, Madrid, Spain; 21 Hospital Provincial Castellón, Castellón de la Plana, Spain; 22 Complejo Hospitalario de Jaén, Jaén, Spain; 23 Hospital del Ferrol, Ferrol, Spain; 24 Hospital Regional Universitario, Málaga, Spain; 25 Hospital Miguel Servet, Zaragoza, Spain

**Keywords:** Metastatic pancreatic adenocarcinoma, Gemcitabine, Nab-paclitaxel, Real-life, First-line chemotherapy, Survival

## Abstract

**Background:**

Treatment with nab-paclitaxel plus gemcitabine increases survival in patients with metastatic pancreatic cancer. However, the assessment of treatment efficacy and safety in non-selected patients in a real-life setting may provide useful information to support decision-making processes in routine practice.

**Methods:**

Retrospective, multicenter study including patients with metastatic pancreatic cancer, who started first-line treatment with nab-paclitaxel plus gemcitabine between December 2013 and June 2015 according to routine clinical practice. In addition to describing the treatment pattern, overall survival (OS) and progression-free survival (PFS) were assessed for the total sample and the exploratory subgroups based on the treatment and patients’ clinical characteristics.

**Results:**

All 210 eligible patients had a median age of 65.0 years (range 37–81). Metastatic pancreatic adenocarcinoma was recurrent in 46 (21.9%) patients and de novo in 164 (78.1%); 38 (18%) patients had a biliary stent. At baseline, 33 (18.1%) patients had an ECOG performance status ≥2. Patients received a median of four cycles of treatment (range 1–21), with a median duration of 3.5 months; 137 (65.2%) patients had a dose reduction of nab-paclitaxel and/or gemcitabine during treatment, and 33 (17.2%) discontinued treatment due to toxicity. Relative dose intensity (RDI) for nab-paclitaxel, gemcitabine, and the combined treatment was 66.7%. Median OS was 7.2 months (95% CI 6.0–8.5), and median PFS was 5.0 months (95% CI 4.3–5.9); 50 patients achieved either a partial or complete response (ORR 24.6%). OS was influenced by baseline ECOG PS, NLR and CA 19.9, but not by age ≥ 70 years and/or the presence of hepatobiliary stent or RDI < 85%. All included variables, computed as dichotomous, showed a significant contribution to the Cox regression model to build a nomogram for predicting survival in these patients: baseline ECOG 0–1 vs. 2–3 (*p* = 0.030), baseline NLR > 3 vs. ≤ 3 (*p* = 0.043), and baseline CA 19.9 > 37 U/mL vs. ≤37 U/mL (*p* = 0.004).

**Conclusions:**

Nab-Paclitaxel plus gemcitabine remain effective in a real-life setting, despite the high burden of dose reductions and poorer performance of these patients. A nomogram to predict survival using baseline ECOG performance status, NLR and CA 19.9 is proposed.

## Background

Pancreatic adenocarcinoma, the most common form of pancreatic cancer, is currently the fourth cause of cancer-related mortality in Europe and the United States, with a 5-year survival rate in the range of 6–10% [1–4]. This has been attributed, among other causes, to the premature vascular, lymphatic and perineural spread of these tumors, which makes that 85% of patients present disseminated disease at diagnosis. Only between 15 and 20% of tumors are resectable and, within these, 50 to 86% will experience local failure despite curative resection, with a resulting 5-year survival rate of 10–20% [5].

Gemcitabine has been the standard first-line treatment for advanced pancreatic cancer for 15 years, and it is associated with median overall survivals (OS) ranging from 5.6 to 6.8 months [6]. Its combination with a wide range of other agents such as capecitabine, oxaliplatin, cisplatin, irinotecan, and erlotinib has unfortunately shown little impact on survival in this population [6, 7]. Nonetheless, two combination regimens, FOLFIRINOX (folinic acid, fluorouracil, irinotecan, oxaliplatin) and more recently, nab-paclitaxel plus gemcitabine, have been associated with a median OS of 11.1 months and 8.5 months in the ACCORD4/PRODIGE11 and MPACT phase III clinical trials, respectively [8, 9]. These encouraging results have set a new standard and international guidelines now recommend FOLFIRINOX and nab-paclitaxel plus gemcitabine as first-line treatments in patients with metastatic pancreatic cancer [10, 11].

A questionnaire-based study showed that clinicians are likely to adhere to the selection criteria of these trials, being the patient’s performance status one of the most influential factors in the decision-making process in the real-life setting [12]. As randomized trials have strict selection criteria, particularly regarding performance status and age [8, 9], reported data might not capture the scenario faced in the real-life setting, with non-selected patients. A prospective registry-based study showed that fewer than half of the patients treated in routine clinical practice would have been eligible for the PRODIGE or MPACT trials and that meeting the eligibility criteria for either trial is associated with longer survival [13].

Clinicians are increasingly interested in obtaining real-life data from daily practice, which completes the clinical picture of randomized trials. As such, observational trials can provide useful information for clinicians to support decision-making processes in their routine practice, especially when treating patient groups underrepresented in clinical trials, as are elderly patients and those with poorer performance status [14, 15]. In line with this unmet need, Ellenrieder et al. highlighted the importance of real-life data regarding first-line regimens in metastatic pancreatic cancer to select the most suitable treatment for each patient [7]. The aim of the ANICE study was to assess the effectiveness and tolerability of gemcitabine and nab-paclitaxel as first-line therapy for metastatic pancreatic cancer in a real-life setting.

## Methods

### Study design and patients

The ANICE trial was an observational, retrospective, multicenter study focused on patients with metastatic pancreatic adenocarcinoma (recurrent or de novo) treated according to routine clinical practice at 20 Spanish hospitals between December 2013 and June 2015. All adult patients (≥ 18 years) with measurable metastatic disease at baseline in at least one dimension (per Response Evaluation Criteria in Solid Tumors [RECIST], version 1.1, [16]) who received at least one dose of nab-paclitaxel (Abraxane®, Celgene Europe Limited) plus gemcitabine as first-line chemotherapy were included. Data were obtained from clinical medical records with a cut-off date of 16 March 2017. All patients provided written informed consent. The study protocol was approved by the local independent ethics committee, and was conducted in accordance with the Spanish personal data protection law (LOPD 15/1999).

### Variables and endpoints

Variables collected from the patient’s medical records included demographic data (sex and age), clinical data, characteristics of the metastatic disease and treatment. Among the registered clinical and disease characteristics were performance status (PS) (ECOG and Karnofsky scales), relevant comorbidities, initial diagnosis, number and localization of metastases, time between diagnosis of the primary tumor and recurrence, presence of a hepatobiliary stent, serum bilirubin, neutrophil/lymphocyte ratio (NLR), and CA 19.9 antigen levels. Treatment characteristics included concomitant treatments, relative dose intensity (RDI) for nab-paclitaxel plus gemcitabine (i.e., per the summary of product characteristics), treatment duration, cause of treatment discontinuation, number of cycles, and dose reductions and interruptions. Decisions regarding the initial dose and subsequent dose reductions were made at physicians’ discretion, based on patient’s PS and toxicity, and following the routine practice of each participating sit.

The primary objective was to describe the treatment pattern in terms of extent of exposure and reductions of treatment with nab-paclitaxel plus gemcitabine in real-life clinical practice. Secondary objectives included objective response rate (ORR), progression-free survival (PFS) defined as the time from the start of treatment to disease progression or all-cause death, overall survival (OS) defined as the time from the start of treatment to death from any cause, and the 12-month survival rate defined as the percentage of patients alive at 12 months after starting treatment. Safety was assessed in terms of adverse events (AE), coded according to the preferred term of the Medical Dictionary for Regulatory Activities (MedDRA), and graded according to the National Cancer Institute-Common Toxicity Criteria (version 4.0) [17].

### Statistical analyses

Given the descriptive nature of the statistical analyses, the sample size was calculated based on the confidence interval (CI) of the 12-month survival rate. Considering the 12-month survival rate previously reported by Goldstein et al. [18], and assuming that nearly 20% of patients present with recurrent metastatic cancer, a sample of 225 patients was deemed necessary to estimate a 12-month survival rate of 30% with a ± 6% precision and a 95% CI.

Categorical variables were summarized as frequencies and percentages, and quantitative variables as the mean and standard deviation (SD) and/or median and interquartile range (IQR). The quantitative variables NLR and CA 19.9 were transformed into dichotomous variables with the cut-offs values of 3 and 37 U/mL, respectively. Categorical values were compared using the Fisher’s exact test or the chi-square test when the requirements for a Fisher’s exact test could not be assumed. Quantitative values were compared using the T-test, ANOVA test, and their non-parametric counterparts, the Wilcoxon and Kruskal-Wallis tests. OS and PFS curves were plotted using the Kaplan-Meier estimate, and compared according to selected parameters using the Log-rank test and the Cox regression model. The significance threshold for all bivariate analyses was set at a two-sided α = 0.05. All factors showing a significant influence on OS in the bivariate analysis were included in a Cox regression model as dichotomous variables to build a nomogram for predicting OS in real-life practice. The obtained hazard ratios (HR) were used to associate each variable with a survival score. In addition to the predicted survival, a probability for 3-, 6-, 12, and 18-month survival was estimated. Based on the range of final scores resulting from the possible combinations of variables, low-, medium-, and high-risk groups were defined. All analyses were performed using the statistical package SAS system for Windows version 9.4.

## Results

### Patient characteristics

Of the 216 patients recruited, 6 were excluded for one or more of the following reasons: not initiating combined treatment with gemcitabine plus nab-paclitaxel (*n* = 4), absence of measurable disease at baseline per RECIST (*n* = 2), and being enrolled in a clinical trial (*n* = 1). Table 1 summarizes the demographic and clinical characteristics of study patients. The 210 eligible patients had a median age of 65.0 years (range 37–81). Metastatic pancreatic adenocarcinoma was recurrent in 46 (21.9%) patients and de novo in 164 (78.1%). For patients with recurrent metastatic disease, the primary tumor was resectable in 38 cases (82.6%), borderline in 3 (6.5%), and locally advanced unresectable in 5 (10.9%), and median time to recurrence was 11.0 months. Assessment of eligibility for the MPACT and ACCORD4/PRODIGE11 trials could be assessed in 78 (37.1%) and 172 (81.9%) patients, respectively, with 37 meeting the criteria for entering the MPACT trial and 109 the ACCORD4/PRODIGE11 trial.

**Table 1:** Demographic and clinical characteristics of study patients

	Number^a^	Overall	Recurrent	De novo
Demographic characteristics				
Age, *n (%)*	210			
< 65 years		99 (47.1%)	22 (47.8%)	77 (47.0%)
65–69 years		58 (27.6%)	8 (17.4%)	50 (30.5%)
≥ 70 years		53 (25.2%)	16 (34.8%)	37 (22.6%)
Sex, *n (%)*	210			
Males		127 (60.5)	33 (71.7)	94 (57.3)
Females		83 (39.5)	13 (28.3)	70 (42.7)
Clinical characteristics				
Weight loss > 10%, *n (%)*	208	81 (38.9)	9 (20.5)	72 (43.9)
ECOG PS, *n (%)*	182			
0–1		149 (81.9)	28 (77.8)	121 (82.9)
2–3		33 (18.1)	8 (22.2)	25 (17.1)
Karnofsky PS, *n (%)*	55			
< 70		3 (5.5)	–	3 (6.8)
70–80		33 (60.0)	7 (63.6%)	26 (59.1%)
90–100		19 (34.5)	4 (36.4%)	15 (34.1%)
Common comorbidities, *n (%)*	210			
Hypertension		56 (26.7)	8 (17.4)	48 (29.3)
Diabetes		26 (12.4)	4 (8.7)	22 (13.4)
Dyslipemia		29 (13.8)	6 (13.0)	22 (13.4)
Hepatobiliary stent, *n (%)*	209	38 (18.2)	3 (6.5)	35 (21.5)
Platelet count, *median (IR)*	178	231.0 (172.0, 318.0)	207.0 (170.0, 303.0)	237.0 (181.0, 318.0)
Bilirubin (mg/dL), *median (IR)*	175	0.70 (0.50, 1.00)	0.57 (0.50, 0.80)	0.79 (0.50, 1.10)
NLR, *n (%)*	170			
> 3		91 (53.5)	18 (52.9)	73 (53.7)
≤ 3		79 (46.5)	16 (47.1)	63 (46.3)
CA 19.9, *n (%)*	173			
> 37 U/mL		142 (82.1)	32 (82.1)	110 (82.1)
≤ 37 U/mL		31 (17.9)	7 (17.9)	24 (17.9)
Number of metastatic sites, *n (%)*	210			
1–3		205 (97.6)	46 (100.0)	159 (97.0)
> 3		5 (2.4)		5 (3.0)
Concommitant treatment, *n (%)*	210			
Analgesics		76 (36.2)	12 (26.1)	63 (38.4)
Corticosteroids		25 (11.9)	4 (8.7)	20 (12.2)

*IR* Interquartile range (percentile 25, percentile 75)

^a^number of evaluable patients (no-missing)

### Treatment characteristics and outcome

Table 2 summarizes treatment characteristics. Median time from diagnosis to treatment start was 28 days. Patients received a median of 4 cycles (range 1–21) of treatment, with a median treatment duration of 3.5 months. Sixty-eight patients (32%) started treatment with dose reduction for either nab-paclitaxel, gemcitabine or both drugs. Table 3 summarizes the baseline characteristics of the 68 patients with dose reduction and treatment start. The median RDIs were 66.7% for nab-paclitaxel, gemcitabine, and the combined treatment. Overall, 137 patients (65.2%) had a dose reduction in nab-paclitaxel and/or gemcitabine during treatment. Thirty-four (17%) patients received ≤30 days of treatment, mainly due to toxicity (*n* = 11) or disease progression (*n* = 10). There were no significant differences between patients receiving ≤30 days of treatment and those receiving > 30 days in terms of baseline clinical characteristics including performance status, the presence of a hepatobiliary stent, NLR, CA 19.9 and bilirubin levels, or weight loss of more than 10%.

**Table 2:** Treatment characteristics

	No. (%)
Started treatment with dose reduction, *n (%)*	
Only gemcitabine	1 (0.5)
Only nab-Paclitaxel	41 (19.5)
Both	26 (12.4)
Dose reduction during treatment, *n (%)*	
Nab-Paclitaxel	91 (43.3)
Gemcitabine	75 (35.7)
Either of the two drugs	96 (45.7)
Received ≤30 days of treatment, *n (%)*	34 (16.5)
Reasons for treatment discontinuation^a^, *n (%)*	
Progression	134 (69.8)
Toxicity	33 (17.2)
Death	24 (12.5)
Patient’s request	7 (3.6)

^a^A patient could have more than one reason for treatment discontinuation

**Table 3:** Demographic and clinical characteristics of patients who started treatment with dose reduction

Demographic characteristics		
Age, *n (%)*	(*n* = 68)	
< 65 years		33 (48.5%)
65–79 years		14 (20.6%)
≥ 70 years		21 (30.9%)
Sex, *n (%)*	(*n* = 68)	
Males		41 (60.3%)
Females		27 (39.7%)
Clinical characteristics		
Weight loss > 10%, *n (%)*	(*n* = 66)	18 (27.3%)
ECOG PS, *n (%)*	(*n* = 62)	
0–1		46 (74.2%)
2-Mar		16 (25.8%)
Karnofsky PS, *n (%)*	(*n* = 23)	
< 70		–
70–80		13 (56.5%)
90–100		10 (43.5%)
Common comorbidities, *n (%)*	(*n* = 68)	
Hypertension		15 (22.1%)
Diabetes		8 (11.8%)
Dyslipemia		4 (5.9%)
Hepatobiliary stent, *n (%)*	(*n* = 68)	10 (14.7%)
Platelet count, *median (IR)*	(*n* = 57)	252.0 (172.0, 339.0)
Bilirubin (mg/dL), *median (IR)*	(*n* = 58)	0.70 (0.51, 1.10)
NLR, *n (%)*	(*n* = 55)	
> 3		29 (52.7%)
≤ 3		26 (47.3%)
CA 19.9, *n (%)*	(*n* = 62)	
> 35		52 (83.9%)
≤ 35		10 (16.1%)
Number of metastatic sites, *n (%)*	(*n* = 68)	
1–3		67 (98.5%)
> 3		1 (1.5%)
Concommitant treatment, *n (%)*	(*n* = 68)	
Analgesics		22 (32.4%)
Corticosteroids		9 (13.2%)

*IR* Interquartile range (percentile 25, percentile 75)

^a^number of evaluable patients (no-missing)

At the time of data collection, median follow-up was 7.2 months (IQR 3.5–13.3). A total of 193 patients (91.9%) had died, with a 12-month survival rate of 30.1%. Median OS was 7.2 months (95% CI 6.0–8.5), and median PFS 5.0 months (95%CI 4.3–5.9). Among 203 patients eligible for response, 50 patients achieved either a partial or complete response (ORR 24.6%), and 72 patients (35.5%) a stable disease. Ninety-seven patients (46.9%) received further treatment lines, most of whom (*n* = 63, 64.9%) had one or two lines of treatment (*n* = 25, 25.8%).

### Safety

Grade ≥ 3 treatment-related AEs were reported in 78 patients (37.1%), the most common being neutropenia, thrombocytopenia, and fatigue (Table 4). One treatment-related event of paralytic ileus led to treatment interruption and death.

**Table 4:** Common treatment-related adverse events (> 1% of patients overall) of grade 3. No. (%)

	Overall (*n* = 210)	< 70 years (*n* = 157)	≥ 70 years (*n* = 53)
Hematological toxicities			
Neutropenia	38 (18.1)	33 (21.0)	5 (9.4)
Thrombocytopenia	13 (6.2)	9 (5.7)	4 (7.5)
Anemia	7 (3.3)	2 (1.3)	5 (9.4)
Febrile neutropenia	4 (1.9)	2 (1.3)	2 (3.8)
Non-hematological toxicities			
Fatigue	13 (6.2)	10 (6.4)	3 (5.7)
Vomiting	3 (1.4)	1 (0.6)	2 (3.8)
Colangitis	3 (1.4)	3 (1.9)	–
Neurotoxicity	3 (1.4)	1 (0.6)	2 (3.8)
Peripheral neuropathy	5 (2.4)	3 (1.9)	2 (3.8)
Alopecia	9 (4.3)	6 (3.8)	3 (5.7)

Patients aged < 70 years received a median of 5 cycles of combined treatment and patients aged ≥70 years received a median of 3. Of 53 elderly patients (i.e., aged ≥70 years), 38 (71.7%) experienced at least one treatment-related AE, compared to 78.1% in the overall population, with similar profile of grade ≥ 3 events (Table 4). No significant differences were found in the frequency of treatment-related AEs of grade 3 or higher between patients aged ≥70 years and <  70 (39.6% vs. 36.3%; *p* = 0.789).

### Prognostic factors

**Fig. 1:**
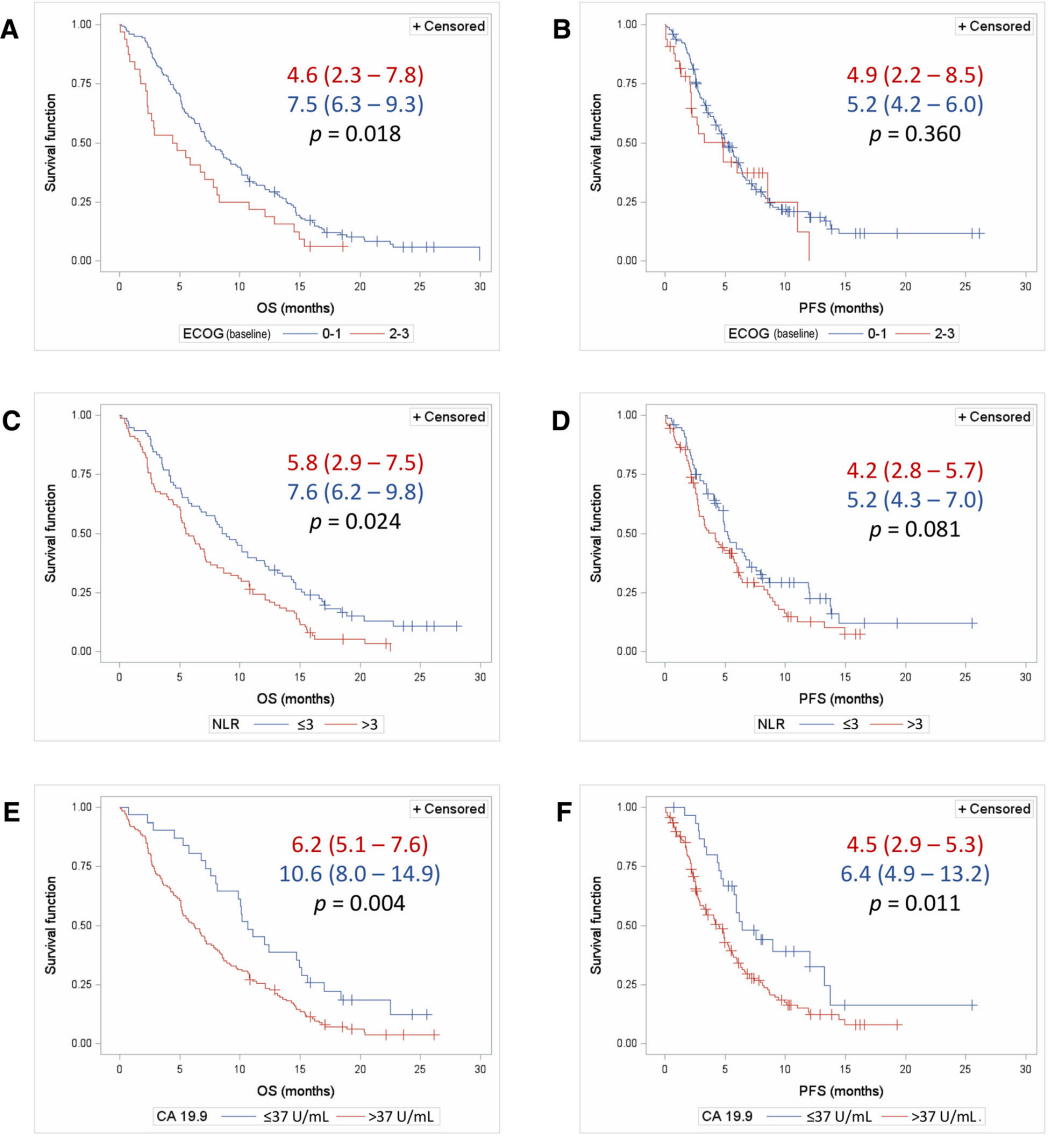
Overall survival (**a**, **c** and **e**) and progression-free survival (**b**, **d** and **f**) depending on baseline ECOG (**a** and **b**), NLR (**c** and **d**) and CA 19.9 (**e** and **f**). Survival is presented as median (95% CI); *p*-values correspond to the Log-rank test for inter-curve differences

Analysis of the influence of baseline characteristics on survival showed that only ECOG, NLR, and CA 19.9 significantly influenced OS, PFS (Fig. 1), and/or 12-month survival. Baseline CA 19.9 had a significant influence on the three analyzed outcomes: patients with CA 19.9 ≤ 37 U/mL had longer OS (*p* = 0.004) (Fig. 1e), PFS (*p* = 0.011) (Fig. 1f), and a higher 12-month survival rate (45.2% vs. 24.8%; *p* = 0.030). On the other hand, patients with baseline NLR ≤ 3 had longer OS (*p* = 0.024) (Fig. 1c) and a higher 12-month survival rate than those with NLR > 3 (38% vs. 23.3%; *p* = 0.045) but showed similar PFS (Fig. 1d). Patients with a baseline ECOG PS of 0 or 1 had longer OS than those with an ECOG PS of 2 or 3 (*p* = 0.018) (Fig. 1a), although this trend was not observed in PFS (Fig. 1b). Likewise, the 12-month survival for patients with ECOG PS 0 or 1 and 2 or 3 was 31.1 and 21.2%, respectively (*p* = 0.297). Patients with stable ECOG (*n* = 68) or ECOG improvement (*n* = 22) during the first three treatment cycles had longer OS than those with a worsening ECOG (*n* = 19), with a median OS of 12.9 months (95% CI 6.7–15.4), 10.6 months (95% CI 8.1–13.3), and 7.1 months (95% CI 4.7–9.2) for ECOG improvement, stable, and worsening, respectively (*p* = 0.020).

**Fig. 2:**
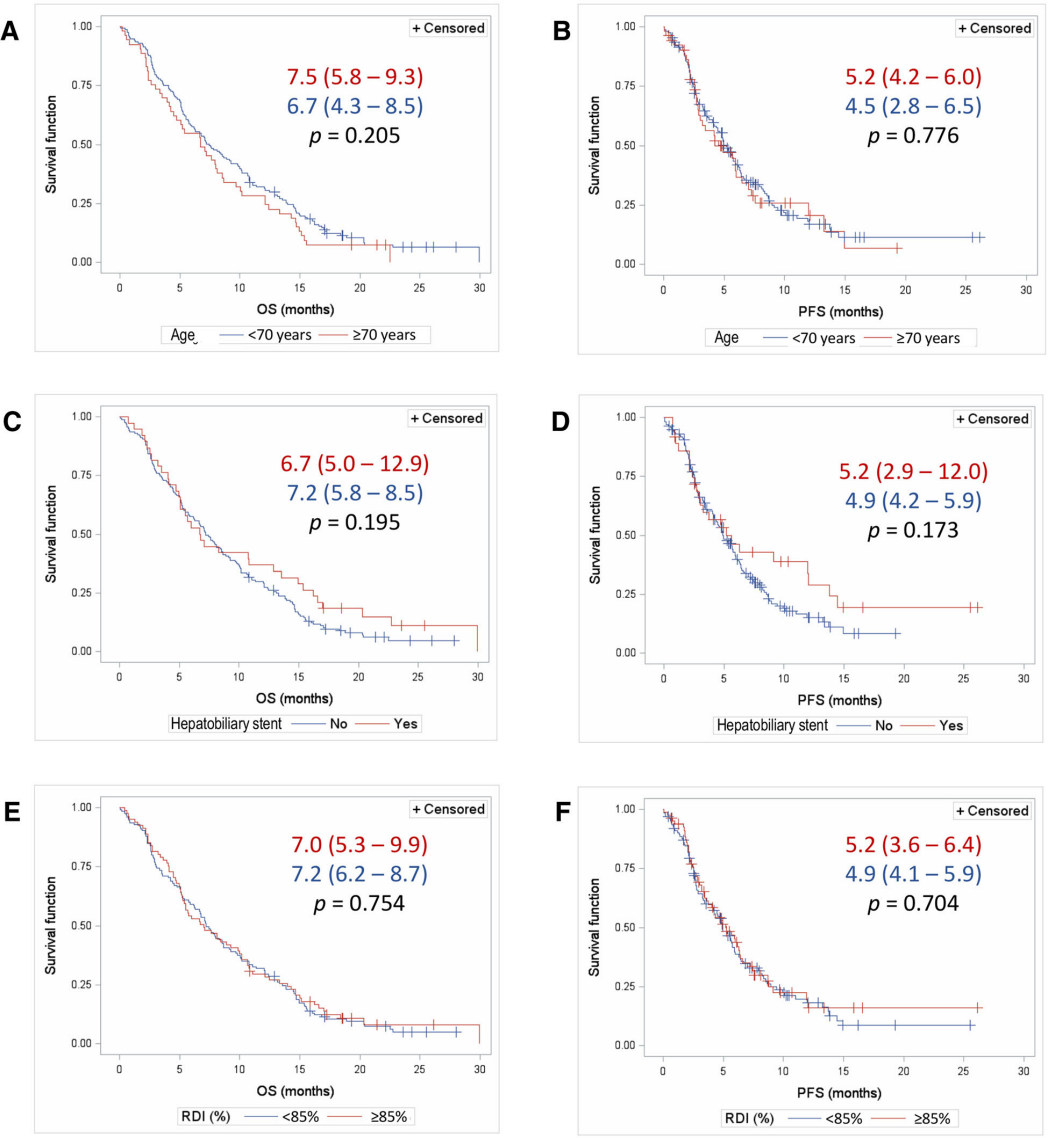
Overall survival (**a**, **c** and **e**) and progression-free survival (**b**, **d** and **f**) depending on baseline age (**a** and **b**), presence of hepatobiliary stent (**c** and **d**), and relative dose intensity (RDI) (**e** and **f**). Survival is presented as median (95% CI); p-values correspond to the Log-rank test for inter-curve differences

Neither age ≥ 70 years, the presence of an hepatobiliary stent, nor an RDI of gemcitabine plus nab-paclitaxel < 85% showed a significant influence on median PFS and OS at 12 months (Fig. 2). The 12-month survival rate was 28.3 and 30.8% for patients aged ≥70 and <  70 years, respectively (*p* = 0.863); 36.8 and 28.8% for patients with and without hepatobiliary stent, respectively (*p* = 0.335); and 28.0 and 31.5% for patients with RDI of the combined treatment ≥85 and < 85%, respectively (*p* = 0.645).

**Fig. 3:**
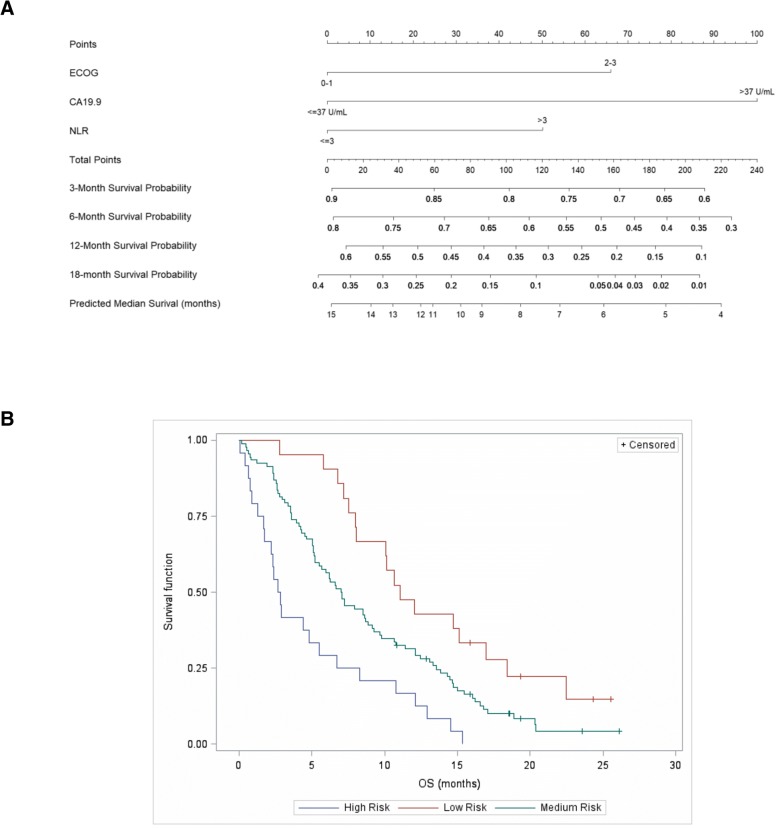
Survival estimate of patients with metastatic pancreatic cancer starting combined treatment with nab-paclitaxel plus gemcitabine in real-life practice. **a** Nomogram for predicting overall survival and the probability of 3-month, 6-month, 12-month in real-life practice. **b** Survival (Kaplan Meier estimate) for low-, medium-, and high-risk groups

Factors significantly influencing OS (ECOG PS, NLR, and CA 19.9) were used to build a nomogram to predict survival of patients with pancreatic adenocarcinoma treated in the real-life setting (Fig. 3a). All included variables, computed as dichotomous, showed a significant contribution to the Cox regression model: baseline ECOG PS 0 or 1 vs 2 or 3 (*p* = 0.030), baseline NLR > 3 vs ≤ 3 (*p* = 0.043), and baseline CA 19.9 > 37 U/mL vs ≤ 37 U/mL (*p* = 0.004) (Fig. 3a). Based on the scale obtained with the nomogram, three risk groups were defined: low-risk group (*n* = 21, 15.1%), medium-risk group (*n* = 93, 66.9%), and high-risk group (*n* = 25, 18.0%). Figure 3b shows the survival curves (Kaplan-Meier estimates for these groups).

## Discussion

In this observational retrospective study including all the patients with metastatic pancreatic cancer treated at the participating centers with first-line nab-paclitaxel plus gemcitabine in a real-life setting, 25% of patients were aged 70 years or more and 18% had a baseline ECOG score of 2 or 3. Furthermore, 32% of patients started combined treatment with a dose reduction of nab-paclitaxel, and 17% of patients discontinued treatment within 30 days of treatment, 33% of them due toxicity. Despite the poor baseline characteristics in our cohort, median OS and PFS were of 7.2 months (95% CI 6.0, 8.5) and 5.0 months (4.3, 5.9), respectively, indicating that this combination is effective in the real-life setting. OS was influenced by the baseline ECOG PS, NLR, and CA 19.9, but not by age ≥ 70 years, the presence of hepatobiliary stent or RDI < 85%.

Randomized controlled trials investigating therapies for metastatic pancreatic cancer have restrictive selection criteria, particularly regarding the patient’s age and performance status [8, 9]. In the case of the pivotal study of the combined treatment (MPACT trial), a Karnofsky index of 70 or more was required [9]. In Spain the preferred scale to assess the performance status in most centers is the ECOG score, thus, in our study, the Karnofsky index could only be assessed in a limited number of patients. However, although the Karnofsky index and the ECOG score lack a linear relationship for direct comparisons between study populations, the fact that 18% of patients had ECOG ≥2 (roughly ≥70 in the Karnofsky index) indicates a trend towards a poorer average performance status in our study population than those in the pivotal MPACT trial. In the case of the ACCORD4/PRODIGE11 study, recruitment was limited to patients under 76 years with ECOG performance status ≤1; [8] based on these criteria alone, nearly 40% of our study patients would have been excluded. In addition to the patients’ baseline characteristics, treatment patterns in real-life practice often differ from those used in pivotal trials. In our study, 66.2% of patients had a dose reduction in either nab-paclitaxel and/or gemcitabine. Overall, the median RDI was 67% for both nab-paclitaxel and gemcitabine, which is well below those reported in the MPACT trial (81 and 75% for nab-paclitaxel and gemcitabine, respectively). Thus, the demographic, clinical and treatment characteristics of this large cohort of real-life patients underscore fundamental differences between RCTs and routine practice settings.

Despite the inclusion of elderly patients, the trend towards a poorer performance status, and adjusted treatment schedule in our study, our results confirmed the effectiveness of combined treatment with gemcitabine plus nab-paclitaxel in the real-life setting. The estimated median OS (7.2 months) was slightly lower than that observed in the MPACT trial (8.5 months) and those reported by Giordano et al. (11 months), Lo Re et al. (9.2 months), and De Vita et al. (10 months) in three series of 118, 37, and 41 real-life patients, respectively [19–21]. Likewise, the median PFS of our cohort (5.0 months) was comparable to that reported in the MPACT trial (5.5 months), but lower than that reported by Giordano et al. (7 months), Lo Re et al. (6.2 months), and of notably Da Vitta et al. (9.2 months). Of note, patients of our cohort had poorer PS than that reported in these studies.

As reported in previous studies, neutropenia was the most common treatment-related adverse event of grade 3 or higher, however its 18% incidence was substantially below that reported in the MPACT trial (38%) [9] and slightly lower than in previous series of real-life patients (20 to 24%) [14, 19, 20]. Remarkably, being aged over 70 years was not associated with a worse toxicity profile in our study, consistent with the trend reported by Giordano et al. in a retrospective study addressing the safety of this treatment in the elderly [14].

In addition to assessing the safety and effectiveness of first-line nab-paclitaxel plus gemcitabine in real-life patients, we also analyzed prognostic factors and their influence on survival in this setting. The influence of the patient’s performance status has been consistently reported by various authors. [18, 19, 21, 22] As in previous analyses of real-life patients, [19, 21] stratifying patients according to the baseline ECOG score of 0–1 or >  1, shows an influence of ECOG on OS. Similarly, the inflammation-based NLR score, identified as a prognostic factor for OS and PFS in patients receiving nab-paclitaxel plus gemcitabine in pivotal studies [18] and real-life practice [20, 21, 23], influenced OS in our patients but did not reach the significance threshold in the PFS analysis. In both localized and metastatic pancreatic adenocarcinoma, a proinflammatory status of the tumor results in worse prognosis, and therefore, influences treatment response and consequently, survival. Finally, the antigen CA 19.9 significantly influenced both OS and PFS with a cut-off of 37 U/mL. Other factors, such as age, the presence of a hepatobiliary stent, and the RDI did not significantly influence either OS or PFS. This finding is particularly controversial for age, which has been identified as a major prognostic factor for all patients with metastatic pancreatic cancer [22], and was subsequently confirmed for patients treated specifically with gemcitabine plus nab-paclitaxel [18]. It is worth noting that the cut-off age considered as a prognostic factor is not homogeneous across studies, and while risk analyses were traditionally based on patients over 60 or 65 years [18, 22], there is increasing interest in investigating patients over 70 years as a risk group in real-life practice [14, 24].

The prognostic factors identified in our cohort (i.e., ECOG PF, NLR, and CA 19.9) allowed us to develop a nomogram for predicting survival of real-life patients treated with first-line nab-paclitaxel plus gemcitabine. A similar tool for predicting survival in real-life patients receiving gemcitabine-based chemotherapy was presented by Hamada et al., and included age, sex, PS, tumor size, and the presence of nodal or distant metastases [25]. As in their analysis, PS carried a notable weight in our nomogram, with patients having an ECOG score of 2 or more dramatically reducing the predicted survival. On the other hand, variables not routinely assessed in standard practice, such as tumor size were not considered. In addition to the nomogram by Hamada et al., Goldstein et al. presented a similar tool based on the cohort of the MPACT trial [26]. The nomogram by Goldstein et al. was similar to ours in terms of the inclusion of performance status and NLR, but included other variables such as albumin, tumor size, and the presence of liver metastasis. Future studies shall validate the proposed nomogram as a tool for predicting survival in real-life patients.

Our results should be interpreted in the context of the intrinsic limitations of retrospective studies. Thus, in addition to the risk of reporting bias associated with observational designs, missing data in the medical records could not be considered for the analysis, leading uneven sample sizes across analyses. The retrospective design also precluded the inclusion of variables not recorded in routine clinical practice in Spain, notably the Karnofsky index, which was reported in very few patients and prevented a direct comparison with the study sample of the pivotal trial MPACT. Another limitation of the retrospective design was the lack of pre-defined criteria for dose reductions, which were established at physician’s discretion, according to the routine practice in each center. Finally, the reduced size of some patient subgroups in the comparative analyses limited the investigation of baseline factors with potential influence on patient survival.

## Conclusions

Our results, obtained from the largest published series of real-world patients with metastatic pancreatic cancer, show that nab-paclitaxel plus gemcitabine remains effective in this setting, despite the high burden of dose reductions and the poorer performance of these patients. Based on the exploratory analysis of prognostic factors, a nomogram was developed to predict survival of patients starting treatment with the combination. It was based on three variables routinely assessed in real-life practice, ECOG performance status, NLR, and CA 19.9.

## Abbreviations

AE: Adverse events
CI: Confidence interval
HR: Hazard ratio
IQR: Interquartile range
NLR: Neutrophil lymphocyte rate
ORR: Objective response rate
OS: Overall survival
PFS: Progression-free survival
PS: Performance status
RDI: Relative dose intensity
RECIST: Response Evaluation Criteria in Solid Tumors
